# NRF2-regulated metabolic gene signature as a prognostic biomarker in non-small cell lung cancer

**DOI:** 10.18632/oncotarget.19349

**Published:** 2017-07-18

**Authors:** Akhileshwar Namani, Qin Qin Cui, Yihe Wu, Hongyan Wang, Xiu Jun Wang, Xiuwen Tang

**Affiliations:** ^1^ Department of Biochemistry, Zhejiang University, Hangzhou 310058, PR China; ^2^ Department of Thoracic Surgery, First Affiliated Hospital, Zhejiang University, Hangzhou 310058, PR China; ^3^ Department of Pharmacology, School of Medicine, Zhejiang University, Hangzhou 310058, PR China

**Keywords:** KEAP1, NRF2, NSCLC, biomarker, metabolism

## Abstract

Mutations in Kelch-like ECH-associated protein 1 (KEAP1) cause the aberrant activation of nuclear factor erythroid-derived 2-like 2 (NRF2), which leads to oncogenesis and drug resistance in lung cancer cells. Our study was designed to identify the genes involved in lung cancer progression targeted by NRF2. A series of microarray experiments in normal and cancer cells, as well as in animal models, have revealed regulatory genes downstream of NRF2 that are involved in wide variety of pathways. Specifically, we carried out individual and combinatorial microarray analysis of KEAP1 overexpression and NRF2 siRNA-knockdown in a KEAP1 mutant-A549 non-small cell lung cancer (NSCLC) cell line. As a result, we identified a list of genes which were mainly involved in metabolic functions in NSCLC by using functional annotation analysis. In addition, we carried out *in silico* analysis to characterize the antioxidant responsive element sequences in the promoter regions of known and putative NRF2-regulated metabolic genes. We further identified an NRF2-regulated metabolic gene signature (NRMGS) by correlating the microarray data with lung adenocarcinoma RNA-Seq gene expression data from The Cancer Genome Atlas followed by qRT-PCR validation, and finally showed that higher expression of the signature conferred a poor prognosis in 8 independent NSCLC cohorts. Our findings provide novel prognostic biomarkers for NSCLC.

## INTRODUCTION

Lung cancer is the most common cancer-related cause of death in the United States and worldwide. Non-small cell lung cancer (NSCLC) is the major type; it accounts for ∼85% of cases and is divided into two major subtypes, squamous cell carcinoma and adenocarcinoma [1, 2]. NSCLC is a complex heterogeneous cancer and identification of its molecular and genomic alterations allows the researchers to discover the clinically useful biomarkers to treat specific patients group by applying personalized medicine. Biomarkers are one of the principal driving forces of human cancers and identification of mutations based specific biomarkers is the great approach to classify the lung cancer patients into different taxa. For instance, epidermal growth factor receptor (EGFR)-positive NSCLC patients were separated into special group in which the patients are treated with EGFR inhibitors as compared with non-EGFR-mutated counterparts. Similarly, lymphoma kinase (ALK) inhibitors are the recently using personalized medicine approaches for ALK-rearranged NSCLC patients group [3, 4]. Thus, there is much scope to identify the novel therapeutic targets for druggable mutations of other proteins in NSCLC. Therefore, in order to explore the potential biomarkers in KEAP1/NRF2 mutated NSCLC, we performed integrated multi-omics approach by using A549 NSCLC cell lines and TCGA lung adenocarcinoma patients data.

The cap‘n’collar type of basic leucine zipper transcription factor, nuclear factor erythroid-derived 2-like 2, also known as NRF2, transactivates genes involved in antioxidant and cytoprotective functions against oxidative insults [5]. Under homeostatic conditions, NRF2 is sequestered in the cytoplasm by binding with its negative regulator Kelch-like ECH-associated protein 1 (KEAP1). KEAP1 acts as a substrate adaptor for the cullin-dependent E3 ligase (CUL3), and the KELCH domains of the KEAP1 heterodimer bind with the N-terminal Neh2 domain of NRF2, leading to the ubiquitination and proteasomal degradation of NRF2 [6].

Under stress conditions, oxidative and electrophilic stress activate NRF2 from KEAP1 retention that leads to the stabilization and subsequent nuclear localization of NRF2 [7, 8]. In the nucleus, NRF2 heterodimerizes with sMAF (small musculoaponeurotic fibrosarcoma) and other transcriptional machinery to activate a battery of antioxidant genes through the antioxidant responsive element (ARE) [9, 10]. Despite the chemopreventive action of NRF2, growing evidence suggests that mutations in KEAP1 and/or NRF2 cause the aberrant activation of NRF2, which leads to oncogenesis and drug resistance in cancer cells [11, 12].

Several line of evidences suggested that, increased NRF2 expression associated with poor prognosis and worse cancer-specific survival in lung cancer patients [13, 14]. In addition, KEAP1 mutant NSCLC patients [15] and patients with methylation at the KEAP1 promoter region [16] had worse prognosis and disease progression as compared with other patients respectively. Other studies have revealed that genomic alterations (mutations/deletions) or epigenetic changes (hypermethylation) in KEAP1 lead to its loss of function, along with overexpression of NRF2 and its downstream genes in lung cancer [17, 18]. Thus, NRF2 has become a potential biomarker and therapeutic target in NSCLC, and its downregulation could pave the way to inhibiting tumor growth and overcoming drug resistance.

A large-scale study, The Cancer Genome Atlas (TCGA), identified ∼17% KEAP1 mutations in lung adenocarcinoma (LUAD) patients [19]. In addition, crucial role of NRF2 regulates genes involved in core carbon metabolism pathways such as the pentose phosphate pathway, the tricarboxylic acid cycle, and glycolysis were well reported [20, 21]. However, so far, comprehensive lists of NRF2 targets that regulate the metabolic gene signature of lung cancer have not been revealed. Reprogramming energy metabolism is considered as one of the hallmark of cancer [22]. Here, we found a comprehensive list of NRF2-regulated genes in A549 NSCLC cells that were principally related to different metabolic functions in LUAD by comparing the transcriptional profiles of KEAP1-overexpressing and NRF2 siRNA knockdown (NRF2-KD) A549 cells. By focusing only on the intersection of genes with significant downregulation in the data from both microarrays with a fold-change cutoff >1.5, we identified 34 common NRF2-regulated genes in A549 cells. Further investigation of the AREs in the promoters of these genes and qRT-PCR results provided evidence that NRF2 directly regulates a substantial number of genes through the ARE. We further identified 12 key genes involved in different metabolic pathways as potential driver genes of NRF2 in KEAP1-altered TCGA LUAD patients and considered them as an NRMGS. Subsequently, we carried out overall survival analysis in NSCLC cohorts and concluded that the coordinated expression of the NRMGS is a critical contributor to KEAP1/NRF2-mediated oncogenesis in NSCLC.

## RESULTS

### Gene expression profiling by microarrays

**Figure 1:**
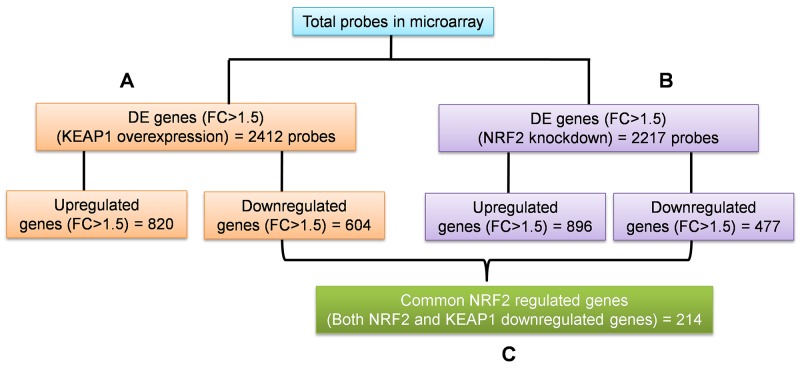
Schematic of the individual and combinatorial analysis of KEAP1-overexpression and NRF2-knockdown microarray data **(A)** KEAP1-overexpression DE genes (FC>1.5) **(B)** NRF2-KD A549 DE genes (FC>1.5) **(C)** The combinatorial analysis of data from A&B. DE, differential expression; FC, fold change.

To identify the regulatory roles of NRF2 in NSCLC, we generated the gene expression profiles of KEAP1-overexpressing and NRF2-KD A549 cells using the GeneChip® PrimeView™ (Affymetrix) Human gene expression array. We systematically analyzed the results obtained from the profiles of both microarrays in three categories (Figure 1). The first two categories comprised individual analyses of KEAP1-overexpressing and NRF2-KD A549 microarrays compared with control (Figure 1A, 1B). The third category was the combinatorial analysis of data from both microarrays (Figure 1C). In all three categories, we considered downregulated genes with a fold-change cutoff >1.5.

### KEAP1-overexpressing *versus* control microarrays

**Figure 2:**
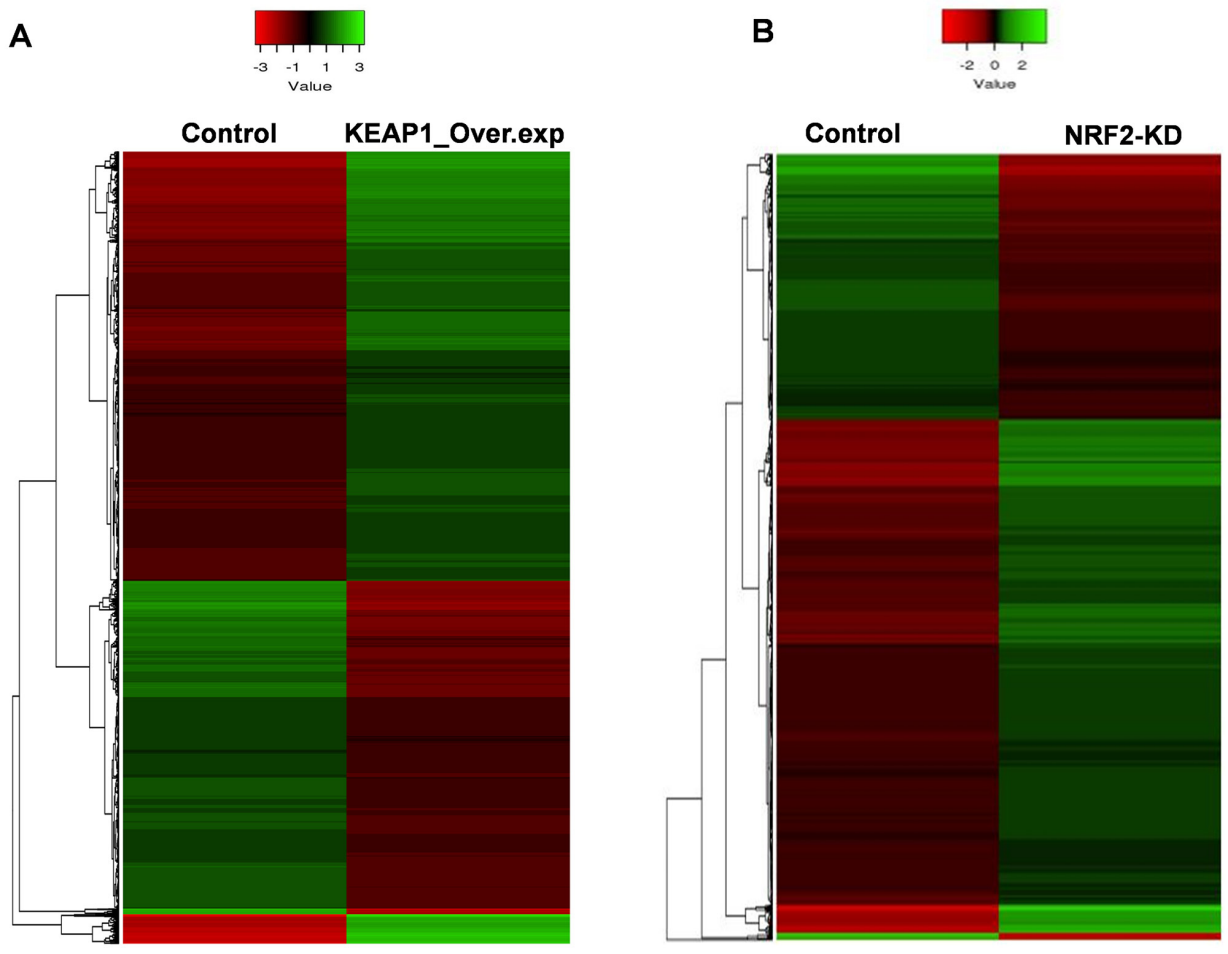
Heatmaps showing the expression profiles and hierarchical clustering of differentially expressed genes in **(A)** KEAP1-overexpression (KEAP1_Over.exp) and **(B)** NRF2-knockdown (NRF2-KD) A549 microarray data compared with control. Green, relatively higher expression; red, relatively lower expression. Heatmap scale bars indicate normalized expression values.

**Figure 3:**
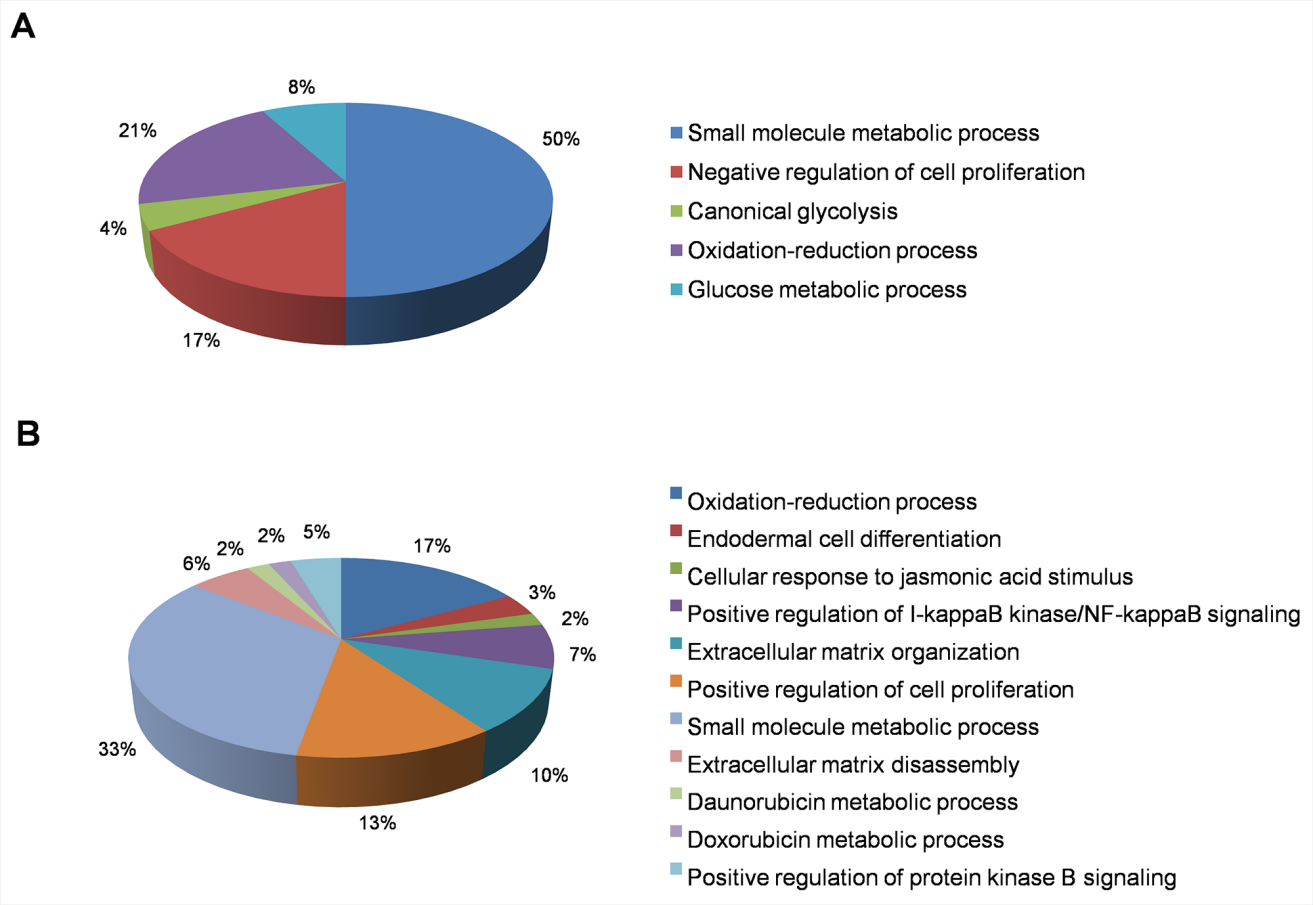
Functional annotation of genes downregulated in KEAP1-overexpressing and NRF2-KD A549 cells Pie charts represent the significantly enriched (*p* <0.001) Gene Ontology Biological Process terms of downregulated genes identified from differentially-expressed genes in **(A)** KEAP1-overexpression and **(B)** NRF2-KD microarrays.

In this category, we found a total of 2412 differentially-expressed probes in the KEAP1-overexpressing A549 cells compared with control (Figure 2A). Of these, 1396 probes encoding 820 genes were upregulated, while 1016 probes encoding 604 genes were downregulated (Figure 1A). We next performed functional annotation of the downregulated genes (Supplementary Table 1) using DAVID (Database for Annotation Visualization and Integrated Discovery) [23]. Gene ontology (GO) analysis (*p* <0.001) of the genes that were downregulated in the KEAP1-overexpressing microarray identified the most significant top-ranked GO-term Biological Process known as “small molecule metabolic process”, demonstrating the specific role of NRF2 in the metabolic functions of cancer cells (Figure 3A). In total, 82 genes involved in various metabolic processes were listed in the category of “small molecule metabolic process” (Supplementary Table 2). Four other GO terms – negative regulation of cell proliferation, canonical glycolysis, oxidation-reduction process, and glucose metabolic process – were also identified in GO analysis, suggesting a role of NRF2 in the regulation of cellular homeostasis and carbon metabolism in NSCLC. Similarly, KEGG pathway analysis (*p* <0.05) also identified key genes involved in metabolic pathways such as carbon metabolism, glycolysis/gluconeogenesis, metabolic pathways, tyrosine metabolism, and others, further supporting the specificity of NRF2 function in metabolism (Supplementary Figure 1A). A full list of genes along with the *p* values of GO and KEGG annotations can be found in Supplementary Table 2.

### NRF2-knockdown *versus* control microarrays

In this category, we found a total of 2217 differentially-expressed probes in the NRF2-KD A549 cells compared with control (Figure 2B). Of these, 1449 probes encoding 896 genes were upregulated, while 768 probes encoding 477 genes were downregulated (Figure 1B). A list of all downregulated genes can be found in Supplementary Table 3. GO analysis (p <0.001) of the downregulated genes identified 11 biological processes: oxidation-reduction process, endodermal cell differentiation, cellular response to jasmonic acid stimulus, positive regulation of I-kappaB kinase/nuclear factor (NF)-kappaB signaling, extracellular matrix organization, positive regulation of cell proliferation, small molecule metabolic process, extracellular matrix disassembly, daunorubicin metabolic process, doxorubicin metabolic process, and positive regulation of protein kinase B signaling (Figure 3B). As anticipated from earlier studies, a total of 33 significant genes related to oxidation-reduction process were downregulated, suggesting a common role of NRF2 in homeostatic functions. Moreover, genes involved in drug metabolism were also downregulated.

Consistent with the KEAP1-overexpressing microarray data, 64 genes involved in various metabolic functions were enriched under the common GO term “small molecule metabolic process” with the significant *p* value-6.30E-04, highlighting the crucial role of NRF2 in cancer metabolism (Supplementary Table 4).

Strikingly, KEGG pathway analysis (*p* <0.05) identified genes involved in the most common NRF2 pathway, metabolism of xenobiotics by cytochrome P450 along with 9 other previously-unknown pathways – insulin resistance, extracellular matrix (ECM)-receptor interaction, thyroid hormone signaling pathway, cyclic guanosine monophosphate-dependent protein kinase G signaling pathway, PI3K-Akt signaling pathway, proteoglycans in cancer, renin secretion, complement and coagulation cascades, and ovarian steroidogenesis – suggesting a diverse and complex role of NRF2 in NSCLC (Supplementary Figure 1B, Supplementary Table 4).

### Combinatorial analysis of KEAP1-overexpression and NRF2-KD microarray data

**Figure 4:**
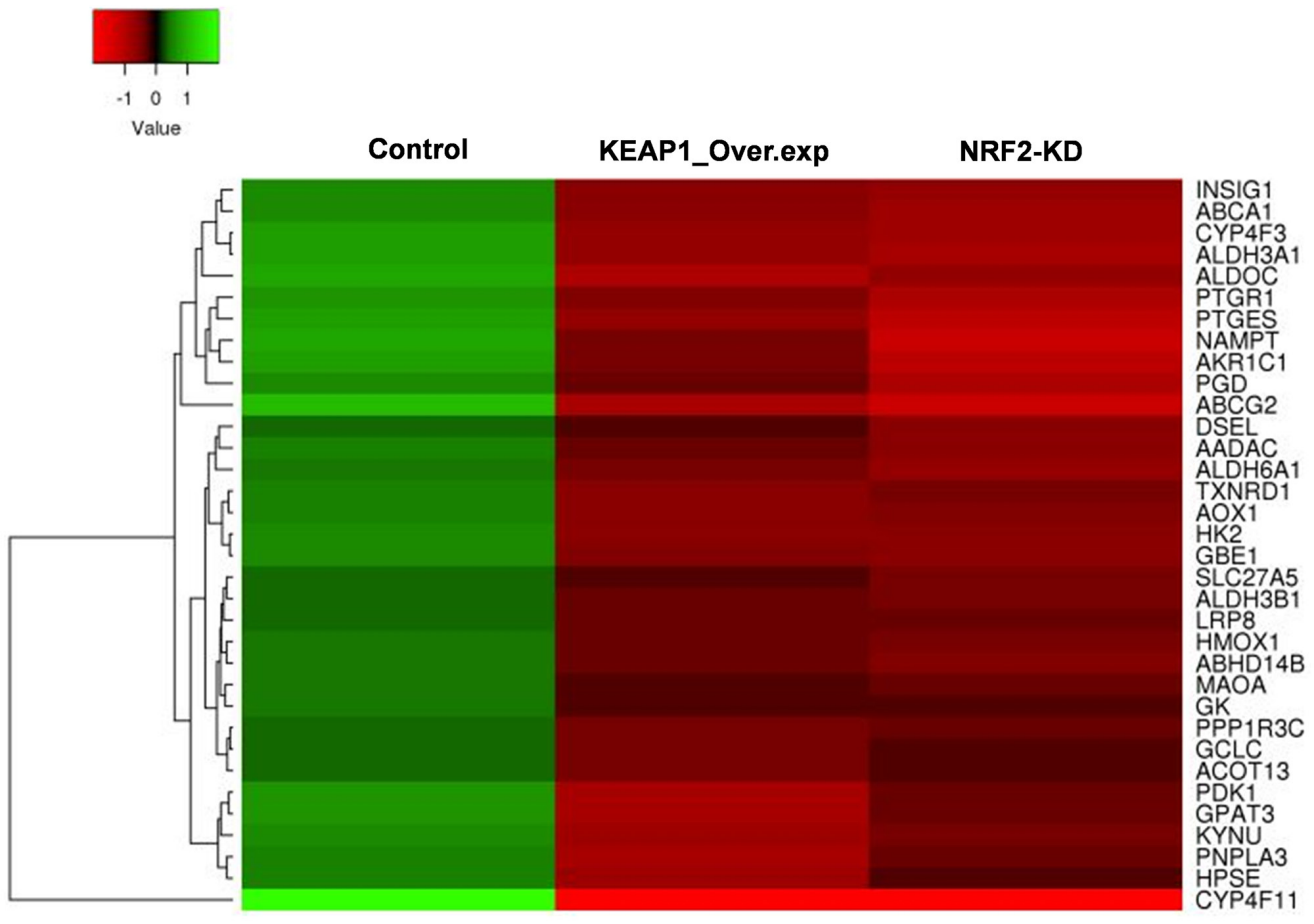
Heatmap showing the expression profiles and hierarchical clusteringof 34 common metabolic genes regulated by NRF2 identified from the integrated analysis of KEAP1-overexpression (KEAP1_Over.exp) and NRF2-knockdown (NRF2-KD) downregulated gene microarray data compared with control.

To identify the target genes of KEAP1/NRF2 in NSCLC, we carried out combinatorial analysis of the downregulated genes (fold change >1.5) in both the KEAP1-overexpressing and NRF2-KD microarray data (Figure 1C). We used the web-based tool ‘Venny’ (http://bioinfogp.cnb.csic.es/tools/venny/) to identify overlapping genes from both datasets to generate a Venn diagram (Supplementary Figure 2). Our analysis revealed ∼24.7% of genes (i.e. 214 overlapping genes) whose expression was significantly associated with NRF2 in both datasets (Supplementary Table 5). We further carried out functional annotation of these overlapping genes using DAVID. As anticipated, GO analysis (*p* ≤0.001) identified genes involved in biological processes: oxidation-reduction process and small molecule metabolic process, as well as negative regulation of cell proliferation, heterotypic cell-cell adhesion, and activation of cysteine-type endopeptidase activity involved in the apoptotic process (Table 1). Interestingly, a cluster of 34 genes involved in small molecule metabolic processes were enriched as the top 2 among other biological processes, suggesting a distinct role of NRF2 in cancer metabolism (Supplementary Table 6). A heat map shows the expression patterns of this cluster of metabolic genes in KEAP1-overexpressing and NRF2-KD A549 cells (Figure 4).

**Table 1:** Functional annotation of both KEAP1 overexpression and NRF2-KD down regulated genes in A549 cells

Term	Count	*p*-value
**GO_Biological Proceess (GO_BP)**		
GO:0055114∼oxidation-reduction process	17	4.39E-04
GO:0044281∼small molecule metabolic process	34	5.73E-04
GO:0008285∼negative regulation of cell proliferation	13	0.001240062
GO:0034113∼heterotypic cell-cell adhesion	4	0.001398769
GO:0006919∼activation of cysteine-type endopeptidase activity involved in apoptotic process	6	0.001591679
**KEGG Pathway**		
hsa00350:Tyrosine metabolism	4	0.011399894
hsa00360:Phenylalanine metabolism	3	0.021483079
hsa00982:Drug metabolism - cytochrome P450	4	0.028043883
hsa00340:Histidine metabolism	3	0.037935313
hsa01100:Metabolic pathways	24	0.052438531

KEGG pathway analysis (*p* ≤0.05) also identified genes involved in different types of metabolic pathway such as tyrosine metabolism, phenylalanine metabolism, drug metabolism-cytochrome P450, histidine metabolism, and metabolic pathways. Altogether, the combinatorial analysis suggested a good level of agreement that KEAP1/NRF2 plays an important role in various metabolic functions in cancer cells (Table 1).

In addition to the functional annotation analysis, a protein-protein interaction network of 214 overlapping downregulated genes obtained by the combinatorial analysis of both microarrays was generated using the Search Tool for the Retrieval of Interacting Genes (STRING) version 10 (http://string-db.org/) database [24]. Interestingly, the GO enrichment results of the network also showed a cluster of proteins related to metabolism. The first top 2 hits of the GO enrichment results consisted of proteins involved in “GO.0009893-positive regulation of metabolic process” and “GO.0031325-positive regulation of cellular metabolic process” with a significant false-discovery rate of 0.00499 (Supplementary Figure 3). Notably, these top 2 GO enrichment results also further support KEAP1/NRF2-mediated metabolic protein regulation in lung cancer.

### Identification of NRF2-ARE sequences by *in silico* analysis

**Figure 5:**
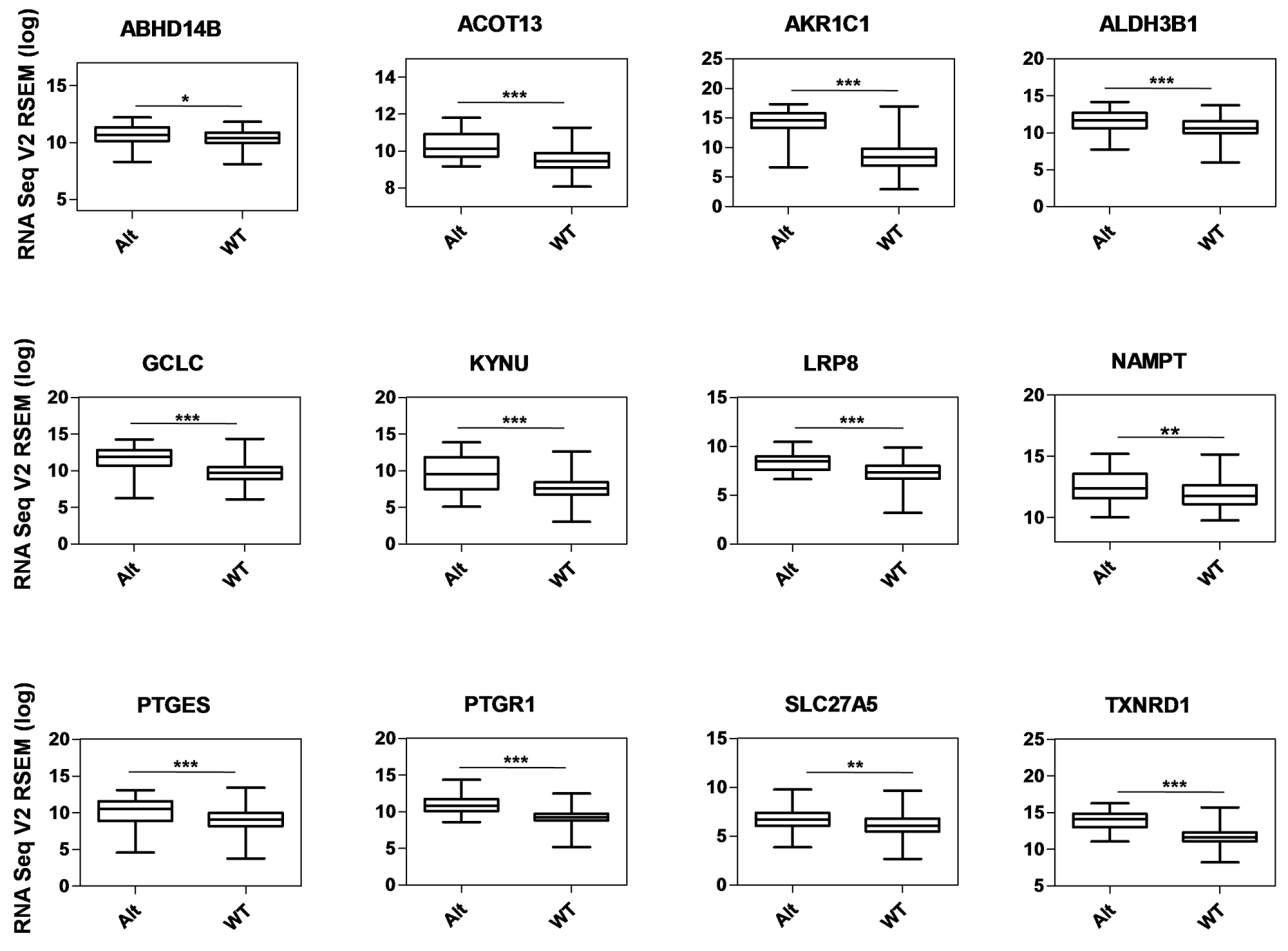
Gene expression analysis of metabolic genes regulated by NRF2 in TCGA LUAD data Box-plots show the mRNA expression levels of metabolic genes regulated by NRF2 in KEAP1-altered (Alt) samples against wild-type (WT) lung adenocarcinoma patients (*p <0.05, **p <0.005, ***p <0.0001). The Y-axis represents the RNA Seq V2 RSEM (log) values from cBioportal.

To identify the consensus ARE sequences in the promoter regions of NRF2-targeted metabolic genes, we performed *in silico* analysis using RSAT (regulatory sequence analysis tools) [25]. For this analysis, we considered a cluster of 34 genes from the GO analysis of overlapping genes, under the term “small molecule metabolic process” (Figure 5). *In silico* analysis identified ARE/ARE-like sequences in 27 of the 34 selected genes (Supplementary Table 7), which suggests that NRF2 directly binds to these genes with its ARE and transactivates their expression in cancer cells. However, we did not find any AREs in the promoter regions of other 7 genes such as AADAC, AOX1, GK, GPAT3, HK2, MAOA, and PGD. Importantly, these 27 ARE genes included some well-known NRF2-regulated genes, such as ABCA1 [26, 27], ABCG2 [28], ALDH3A1 [29], CYP4F3 [30], GBE1 [31], PTGR1 [32], AKR1C1, GCLC, HMOX1, and TXNRD1 [7, 33].

In addition to the known AREs, we identified 17 previously-unknown putative NRF2-regulated genes – ABHD14B, ACOT13, ALDH3B1, ALDH6A1, ALDOC, CYP4F11, DSEL, HPSE, INSIG1, KYNU, LRP8, NAMPT, PDK1, PNPLA3, PPP1R3C, PTGES, and SLC27A5 – which are involved in different metabolic activities in cancer cells.

### Correlation analysis of TCGA LUAD data to identify the KEAP1/NRF2-regulated metabolic gene signature (NRMGS)

A recent comprehensive analysis of LUAD from the TCGA project [19] has revealed the presence of NRF2, KEAP1, and CUL3 mutations in samples from patients. The frequent mutation of these genes causes upregulation of the NRF2 pathway and its gene battery that ultimately leads to tumor progression and drug resistance. Identification of the physiological significance of an NRMGS using clinical correlation analysis with TCGA LUAD data could lead to the discovery of biomarkers in NRF2-overexpressing NSCLC patients.

In order to identify an NRMGS, we used the RNA-Seq: mRNA expression data of a total of 230 TCGA LUAD patients obtained from cBioportal [34]. Of these, 44 samples contained KEAP1 mutations/deletions. However, there were only 4 mutations in each of the NRF2 and CUL3 proteins in all samples. As our aim was to identify a KEAP1/NRF2-regulated NRMGS, we segregated the tumor samples into two types: (1) KEAP1-altered (n = 44): in this category, the samples contained mutations and/or deletions in KEAP1; and (2) wild-type (n = 186): in this category, the samples reflected alterations other than those in KEAP1 (Supplementary Table 8).

We used a list of 27 NRF2 ARE-containing genes obtained from the *in silico* analysis by RSAT for analysis. Of these, 12 genes (ABHD14B, ACOT13, AKR1C1, ALDH3B1, GCLC, KYNU, LRP8, NAMPT, PTGES, PTGR1, SLC27A5, and TXNRD1) showed significantly higher mRNA expression in KEAP1-altered tumors than in wild-type tumors (Figure 5), suggesting that the presence of KEAP1 mutation leads to the overexpression of these genes in LUAD. We then considered this 12-gene cluster as an NRMGS because of the aggressive expression in KEAP1-altered samples.

### Validation of NRMGS candidate genes by qRT PCR

**Figure 6:**
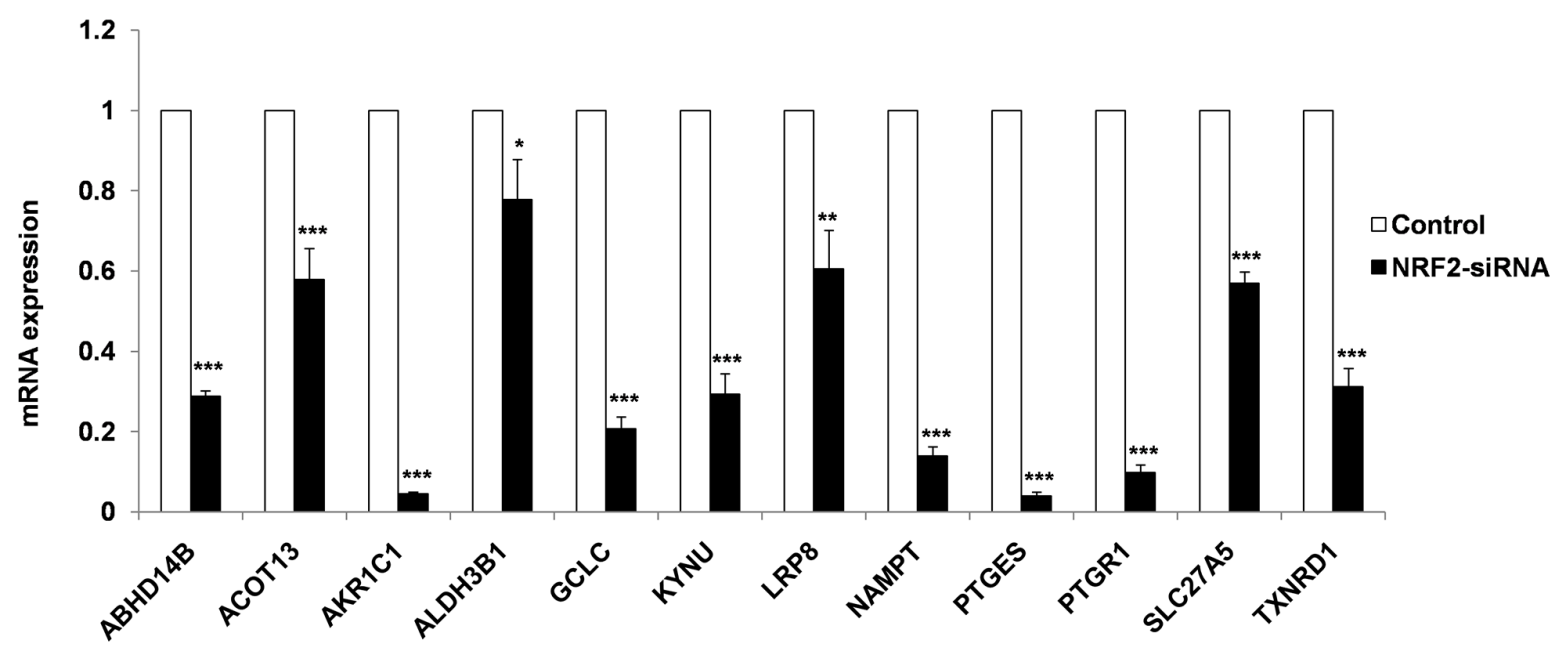
qRT-PCR analysis of NRMGS shows significantly decreased mRNA expression level in NRF2 KD cells as compared with control A549 cells. (*p < 0.05; ** *p* < 0.01, *** *p* < 0.001).

To validate 12 known and novel NRF2-candidate genes listed in NRMGS, we utilized NRF2 KD A549 cell lines for qRT PCR. As a result, all the NRF2-regulated genes listed in NRMGS have shown significantly reduced mRNA expression in NRF2 KD cell lines (*p* < 0.05, t-test), which further support the reliability and reproducibility of our microarray results (Figure 6).

### Higher NRMGS expression is prognostic of poor survival in NSCLC patients

**Figure 7:**
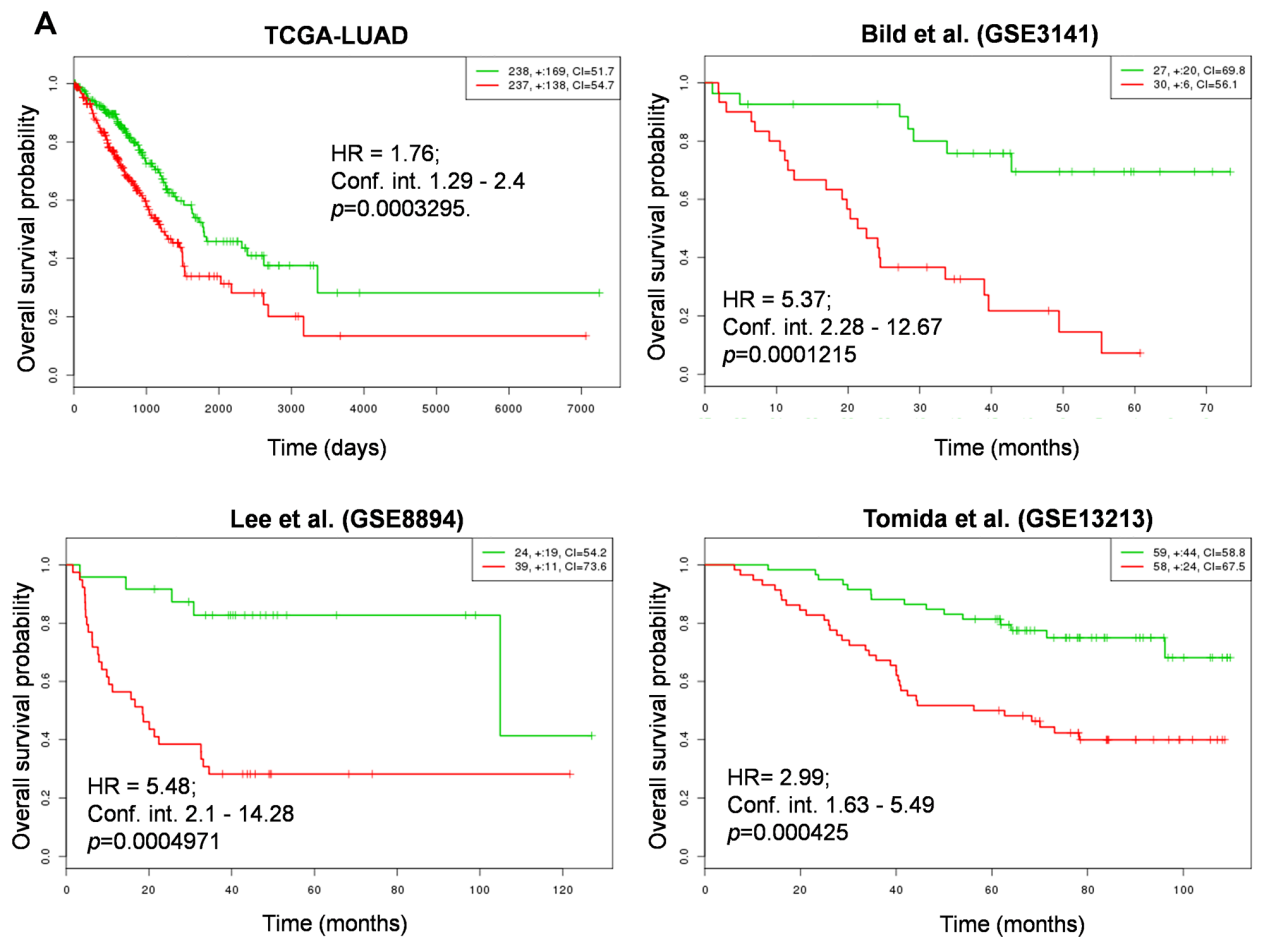

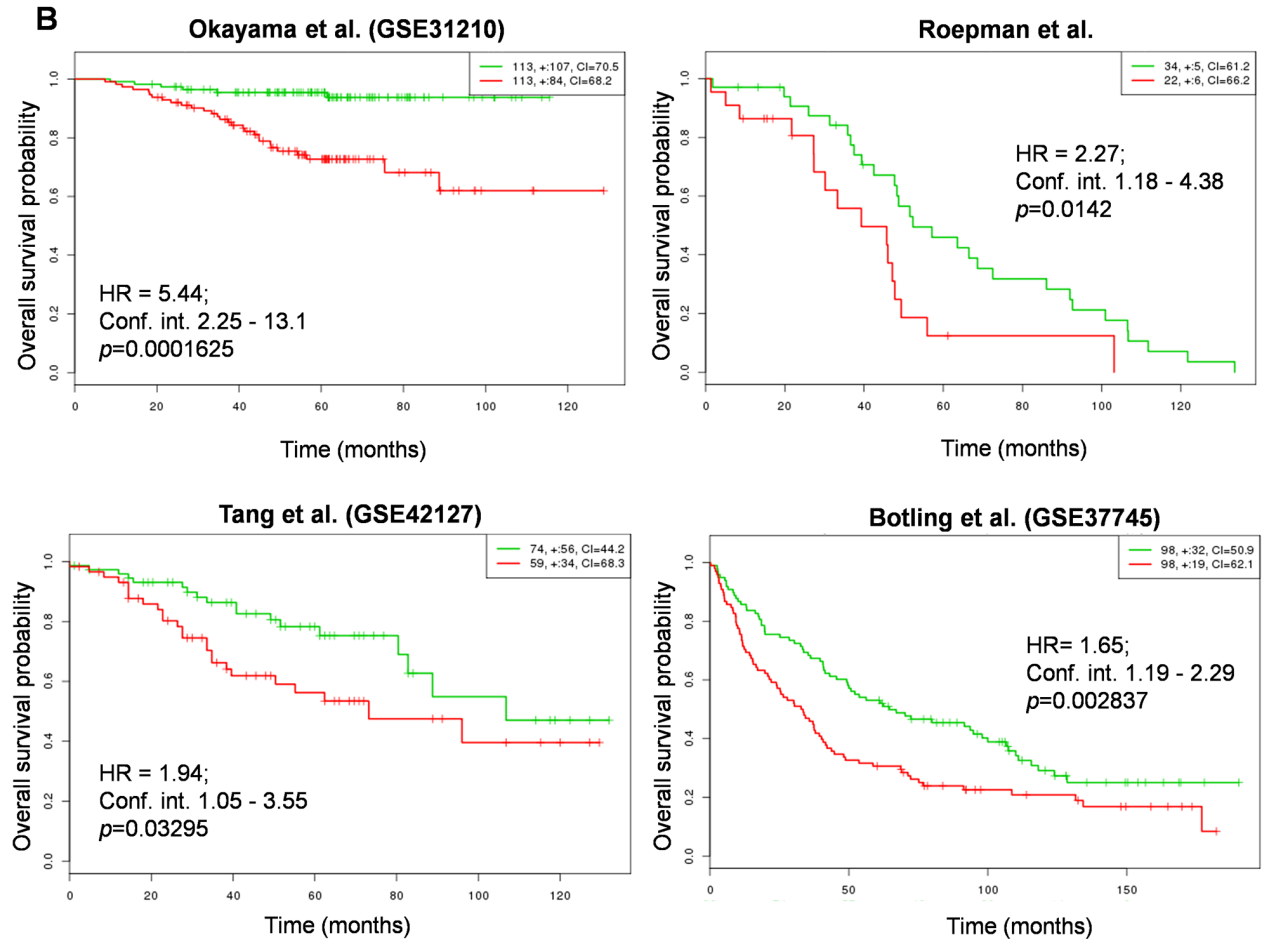
Correlation of NRMGS expression levels and survival in lung cancer patients **(A, B)** Kaplan-Meier survival plots showing that high NRMGS expression is associated with poor survival in 8 independent lung cancer cohorts. Red, high-risk NRMGS curves; green, low-risk NRMGS curves. Insets (upper right), numbers of high- and low-risk samples, number of censored samples marked with +, and concordance index (CI) of each risk group. The X-axis represents time (days or months). The Y-axis shows overall survival probability (HR, hazard ratio; Conf. int., confidence interval).

In order to determine whether NRMGS functions as a prognostic factor for patient survival, we used the online cancer biomarker validation tool SurvExpress [35] in 8 independent lung cancer cohorts. Some cohorts contained data from both lung squamous carcinoma and adenocarcinoma, so we restricted our analysis to patients with adenocarcinoma except Botling et al., which contained both cohorts. The Gene Expression Omnibus (GEO) numbers, total number of samples including high and low risk, hazard ratio, confidence interval, and their corresponding *p* values are listed in Table 2. Strikingly, Kaplan-Meier plots for all cohorts indicated that the patients with higher NRMGS expression showed poorer survival rates (*p <*0.05; Figure 7A, 7B). Thus, overall survival analysis strongly suggested that NRMGS overexpression predicts a poor prognosis for NSCLC patients and can be used as prognostic biomarkers.

**Table 2:** SurvExpress- Cox hazard regression analysis of NRMGS status in 8 independent cohorts

Cohort	GEO Number	No. of samples	High risk	Low risk	Overall/Recurrence-Free Survival		
					HR	CI	*p*-value
TCGA-LUAD	NA	475	237	238	1.76	1.29 - 2.4	0.0003295
Bild et al.	GSE3141	57	30	27	5.37	2.28 - 12.67	0.0001215
Lee et al. *	GSE8894	63	39	24	5.48	2.1 - 14.28	0.0004971
Tomida et al.	GSE13213	117	58	59	2.99	1.63 - 5.49	0.000425
Okayama et al.	GSE31210	226	113	113	5.44	2.25 - 13.1	0.0001625
Roepman et al.	NA	56	22	34	2.27	1.18 - 4.38	0.0142
Tang et al.	GSE42127	133	59	74	1.94	1.05 - 3.55	0.03295
Botling et al.	GSE37745	196	98	98	1.65	1.19 - 2.29	0.002837

NA: Not Applicable, HR: hazard ratio, CI: confidence interval; * Recurrence-Free Survival.

## DISCUSSION

In 2012, Mitsuishi *et al.* first highlighted the role of NRF2 in metabolic reprogramming [20]. They identified 6 genes directly regulated by NRF2 (G6PD, PGD, TKT, TALDO1, ME1, and IDH1) using integrated analysis of ChIP-Seq and microarray data from A549 cells. Then, Singh *et al.* carried out microarray analysis of A549 cells and emphasized key genes involved in carbon metabolism that are mediated by NRF2 [21]. Moreover, recent evidence has shown that NRF2 regulates genes involved in serine and glycine metabolism in NSCLC [36].

Recently, an NRF2-associated molecular signature based on the NRF2-KD A549 cell transcriptome has been identified 50 tumor-associated genes as a set of prognostic biomarkers in NSCLC [37]. To extend their findings, here we used microarray data from both KEAP1-overexpressing and NRF2-KD A549 NSCLC cells to systematically identify an NRF2-regulated gene signature specifically in lung cancer using multi-omics approach. We carried out *in silico* analysis of the promoter regions of known and putative NRF2-regulated genes to identify the ARE sequences. Further, we identified NRF2-regulated metabolic gene signature (NRMGS) contains 12 genes by correlation analysis of RNA-Seq data from TCGA LUAD patients with altered KEAP1. Our results identified known (AKR1C1, GCLC, TXNRD1, [7] and PTGR1 [32]) and novel NRF2 regulated genes (ABHD14B, ACOT13, ALDH3B1, KYNU, LRP8, NAMPT, PTGES, PTGR1, SLC27A5). Furthermore, we carried out qRT-PCR analysis of NRMGS genes in NRF2-KD cell lines. Finally, we validated this NRMGS in 8 independent LUAD cohorts to evaluate its prognostic value in the survival of lung cancer patients and found that higher expression of NRMGS leads to poor survival in LUAD patients.

Interestingly, two of the genes listed in our NRMGS – KYNU (which is involved in tryptophan metabolism) and TXNRD1 – have been reported to be highly upregulated in the gene signature of cell line-derived KEAP1-mutant NSCLC data [38]. TXNRD1 is highly expressed in different human cancers and is considered to be an important target in chemotherapy [29]. Moreover, these two NRF2 target genes are also highly upregulated in data from TCGA patients with mutated KEAP1 and NRF2 in lung squamous cell carcinoma, LUAD, and head and neck squamous cell carcinoma [38]. In addition, the KEAP1 mutant gene signature (27 genes) of Goldstein *et al.* [38] also contains a set of overlapping downregulated genes present in our combinatorial analysis of data from both microarrays: ABCC2, CABYR, CYP4F11, NR0B1, and PGD (Supplementary Table 5). It is noteworthy that a recent publication on a lung cancer biomarker specific to the aldo-keto reductase (AKR) family of proteins states that one of these proteins identified in our NRMGS – AKR1C1 – can be used as a potential biomarker in lung cancer [39]. It is well known that the classic NRF2-regulated gene GCLC (glutamate-cysteine ligase catalytic subunit) is highly expressed in lung cancer and increased GCLC expression is highly correlated with poorer survival. Furthermore, GCLC plays a crucial role in drug resistance and is considered to be a potential drug target in lung and other cancers [40-42]. Interestingly, another known NRF2-regulated gene, PTGR1 (prostaglandin reductase 1), is highly expressed in NSCLC, promotes cancer cell growth through positive regulation of the cyclin-dependent protein kinase complex, and is considered to be a druggable target in lung and hepatocellular carcinoma [43, 44].

Among the other genes in our NRMGS, ACOT13 (also known as thioesterase superfamily member 2) plays a major role in cell proliferation [45] and has been shown to interact with phosphatidylcholine transfer protein to promote mitochondrial fatty-acid oxidation and control glucose utilization in the liver [46]. ALDH3B1 (aldehyde dehydrogenase 3B1) is a key antioxidant enzyme that is highly upregulated in human lung and other tumors [47]. NAMPT (also known as pre-B-cell colony-enhancing factor or visfatin) is a pro-inflammatory adipocytokine which plays a major role in the up-regulation of NF-κB-mediated matrix metalloproteinase expression in lung cancer and promotes the migration and invasion of tumor cells [48]. It has also been shown that NAMPT promotes the epithelial–mesenchymal transition in mammary epithelial cells [49]. A few studies have reported that aberrant expression of PTGES in association with COX-2 contributes to lung tumorigenesis *via* the prostaglandin biosynthetic pathway [50-52]. A recent interesting study on prostate cancer cells has shown that PTGES amplifies epidermal growth factor receptor-driven tumor progression and induces stemness and invasiveness. Surprisingly, in our results, we found PTGES to be a novel NRF2-regulated gene which is highly upregulated in LUAD. Here, we speculate that NRF2 induces stemness in lung cancer cells *via* the transactivation of PTGES gene expression. However, further studies are needed to confirm or deny this speculation.

*In silico* and qRT-PCR analysis clearly supported the notion that, NRF2 directly binds to the ARE sequences located in the promoter regions of ALDH3B1, NAMPT, and PTGES genes and induces their expression in lung cancer. In addition, the specific roles of novel NRF2-targeted genes such as ABHD14B, LRP8, and SLC27A5 in lung cancer need to be investigated.

More interestingly, GO analysis of the NRF2-KD microarray data identified the GO term-positive regulation of I-kappaB kinase/NF-kappaB signaling, which indicates crosstalk between NRF2 and NF-kappaB signaling in NSCLC. The interplay between these pathways involves both transcriptional and translational mechanisms [10]. However, here we hypothesize that NRF2 positively co-regulates a subset of NF-kappaB-downstream genes that further enhance the inflammation and oncogenesis in NSCLC.

Importantly, crosstalk between NRF2 and other signaling pathways also affects the patho-physiological conditions in tumors. For instance, the crosstalk between important transcription factors such as NRF2 and hypoxia-inducible factor (HIF)-1α is a very interesting example in lung cancer. Previous studies have demonstrated that NRF2 increases the stability of HIF-1α by facilitating increased mitochondrial O_2_ consumption [53], and target genes of NRF2 such as HO-1, TRX1, and p62 are potential inducers of HIF-1α [54-56]. It is well reported that HIF-1α plays a major role in tumor proliferation, angiogenesis, migration, and invasion in tumors. KEGG pathway analysis of the NRF2-KD microarray data identified HIF-1α in two major pathways: the thyroid hormone signaling pathway and proteoglycans in cancer (Supplementary Table 4). Thus, NRF2 could function as a positive regulator of HIF-1α activity in tumors and is coordinately involved in tumor progression.

On the other hand, previously-unrecognized cancer-related KEGG pathways such as ECM-receptor interaction, the PI3K-Akt signaling pathway, and proteoglycans in cancer were specifically enriched in the NRF2-KD microarray data. This shows that NRF2-regulated gene expression is highly complex and heterogeneous in lung cancer. For instance, numerous lines of evidence have shown that the genes involved in ECM-receptor interactions are involved in cancer progression by promoting cell growth, metastasis, and the formation of a tumor microenvironment, while also playing major roles in angiogenesis and inflammation [57]. Interestingly, two of the NRF2 target genes involved in ECM-receptor interactions – osteopontin (secreted phosphoprotein 1) and LAMB3 (laminin subunit beta 3) – have been identified as crucial pro-metastatic genes for lung cancer [58]. Similarly, previous observation has shown higher expression of osteopontin in NRF2-mutant lung cancer cells. In addition, NRF2 directly binds to the promoter of osteopontin and regulates its expression in lung cancer cells [59].

Besides, the PI3K-AKT pathway regulates biological processes such as cell proliferation, differentiation, and apoptosis, as well as being involved in oncogenesis, cancer progression, and drug resistance in different cancers [60-62]. Strikingly, our results showed that NRF2 regulates some of the genes involved in the PI3K-AKT pathway, indicating that NRF2 contributes to tumorigenesis and drug resistance *via* activating this pathway. However, further evidence is needed to confirm or deny these results.

In conclusion, taking into account their limitations, NRMGSs can be used as prognostic biomarkers in NSCLC. However, further functional investigation is necessary to identify the mechanisms of regulation. Moreover, our study extends the information about the regulation of a wide variety of metabolic genes and pathways by NRF2 and highlights a strategy for the pharmacological inhibition of NRF2 and its associated gene signature in NSCLC to reduce tumor growth and drug resistance.

## MATERIALS AND METHODS

### Cell cultures and reagents

Unless otherwise stated, all chemicals were from Sigma–Aldrich Co., Ltd. (Shanghai, China). The NSCLC A549 cell line was from the American Type Culture Collection. The A549-derived Nrf2-knockdown cell line, siNrf2-C27, and its control cell line siGFP-C5, were described previously [63]. To generate stable lines overexpressing Keap1, A549 cells were transfected with an mKeap1-pEGFP plasmid expressing mouse Keap-1. After selection in culture medium containing 0.8 mg/ml G418, one clone named mKeap1-C1, which maintained stable overexpression of Keap1 after multiple passages, was chosen for this study. Similarly, a cell line named GFP-c1 was generated after A549 cells were stably transfected with empty pEGFP vector, and served as a negative control.

### RNA isolation and qRT-PCR

Total RNA was prepared using TRIzol reagent (Invitrogen) and the detailed procedures for qRT-PCR were described previously [63]. The primers used in this study were obtained from primer bank [64] except AKR1C1, GCLC [65] and listed in Supplementary Table 8. *p* values <0.05 were considered statistically significant.

### Microarray data analysis

The raw data were analyzed using Agilent GeneSpring GX software (Version 11.0). Normalization of microarray data from KEAP1-overexpressing and NRF2-KD A549 cells along with specific control samples was performed using the Robust Multi-array Average summarization method. The raw microarray data have been submitted to the GEO database with the accession number GSE94393. We used triplicate microarray samples from the KEAP1-overexpressing and NRF2-KD A549 cells, including control samples to minimize off-target effects. The differential expression of genes was carried out in all triplicate samples and the genes that underwent up- or down-regulation were identified using a p-value <0.05 calculated using the t-test. We used a Heatmapper tool [66] to generate heat maps to depict up- and down-regulated genes.

### Functional annotation and protein-protein interaction network analyses

The DAVID web tool [23] was used to identify the functional annotation of downregulated genes (fold-change >1.5) in microarrays from KEAP1-overexpressing, NRF2-KD, and both A549 cells. STRING-v10 was used to identify the protein–protein interaction networks of downregulated genes (fold-change >1.5) obtained from combinatorial analysis of both microarrays [24].

### *In silico* analysis

Putative AREs in metabolic gene promoters were identified using RSAT [25]. The promoter sequence contains -5 kb lengths for 34 selected genes were retrieved from Eukaryotic Promoter Database (EPD) [67]. We then subjected these sequences to RSAT to identify AREs using the string-based pattern matching RSAT program ‘*dna pattern*’ as described previously [68]. Briefly, the DNA patterns RTGASNNNGCR and RTGAYNNNGCR (where R = A or G, S = C or G, Y = C or T, and N = any nucleotide) were used in the query option to identify an exact match of an ARE sequence in the given 5-kb promoter sequences. The DNA patterns designed by Abduallah *et al.* [68] are based on well-known previous publications on the structure of AREs [69-71].

### TCGA gene expression profiles

Data on alteration of the KEAP1 gene (mutation and/or deletion) in The Cancer Genome Atlas (TCGA) lung adenocarcinomas (LUAD) [19] were obtained from the cBioPortal cancer genomic data website [34]. TCGA-LUAD normalized log values of mRNA expression data with RNA-Seq version 2 (RNA Seq V2 RSEM) from 230 patients samples were also downloaded from cBioPortal.

### Survival analysis

Overall survival and Kaplan-Meier analyses were performed using the online multi-cancer biomarker validation tool “SurvExpress” [35]. For the analysis, we used a total number of 1323 patients samples with 8 lung adenocarcinoma datasets present in SurvExpress: TCGA-LUAD (updated June 2016), Bild *et al.* (GSE3141) [72], Lee et al. (GSE8894) [73], Tomida *et al.* (GSE13213) [74], Okayama *et al.* (GSE31210) [75, 76], Roepman *et al.* [77], and Tang *et al.* (GSE42127) [78], Botling *et al.* (GSE37745) [79]. The overall survival analysis procedure was as described elsewhere [35]. Briefly, for all individual cohorts, SurvExpress separated the patient samples into low-risk and high-risk groups based on the prognostic index. Then it performed log-rank tests of input biomarker differences between the two risk groups, estimated the hazard-ratio by fitting a CoxPH using risk group as a covariate, and the concordance indexes by using the “*survival”* package in R (http://cran.r-project.org). For each dataset, we used the average expression score of the gene signature for duplicate probe sets and the original quantile-normalized data as input options for analysis.

### Statistical analysis

The TCGA analysis of LUAD data including altered and wild-type KEAP1 samples was analyzed using GraphPad Prism. Nonparametric Mann–Whitney tests were carried out between two datasets, and *p* values <0.05 were considered statistically significant. All data are expressed as mean ± standard deviation.

## SUPPLEMENTARY MATERIALS FIGURES AND TABLES



















## Abbreviations

AKR: Aldo-keto reductases
ARE: Antioxidant responsive element
CUL3: Cullin-dependent E3 ligase
DAVID: Database for Annotation Visualization and Integrated Discovery
ECM: Extracellular matrix
GO: Gene ontology
HIF1α: Hypoxia-inducible factor 1α
KD: Knockdown
KEAP1: Kelch-like ECH-associated protein 1
KEGG: Kyoto Encyclopedia of Genes and Genomes
LUAD: Lung adenocarcinoma
NRF2: Nuclear factor erythroid 2-related factor
NRMGS: NRF2-regulated metabolic gene signature
NSCLC: Non-small cell lung cancer
PI3K-AKT: Phosphatidylinositol-3-kinase and protein kinase B
RSAT: Regulatory sequence analysis tools
STRING: Search tool for the retrieval of interacting genes
TCGA: The Cancer Genome Atlas
