# Supplementary material for: NRF2-regulated metabolic gene signature as a prognostic biomarker in non-small cell lung cancer
Source: Oncotarget. 2017 Jul 18;8(41):69847–62. doi: 10.18632/oncotarget.19349 (PMC5642521; doi:10.18632/oncotarget.19349)
Supplement: Supplementary file 1 [file oncotarget-08-69847-s001.pdf]

NRF2-regulated metabolic gene signature as a prognostic biomarker in non-small cell lung cancer

SUPPLEMENTARY MATERIALS

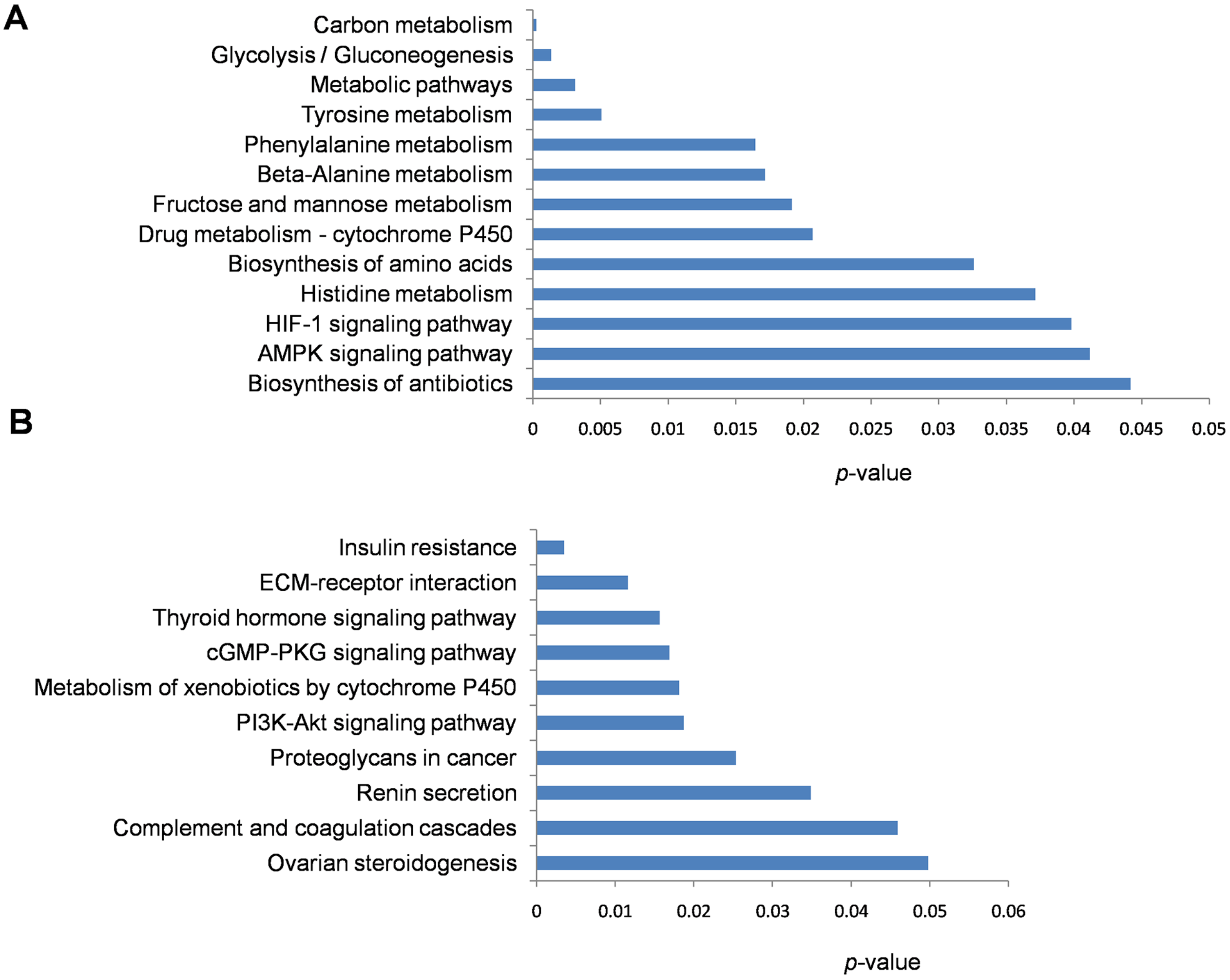

Figure 1: Histograms showing KEGG pathways (p <0.05) of downregulated genes identified from differentially-expressed genes of **(A)** KEAP1-overexpression and **(B)** NRF2-KD microarrays.

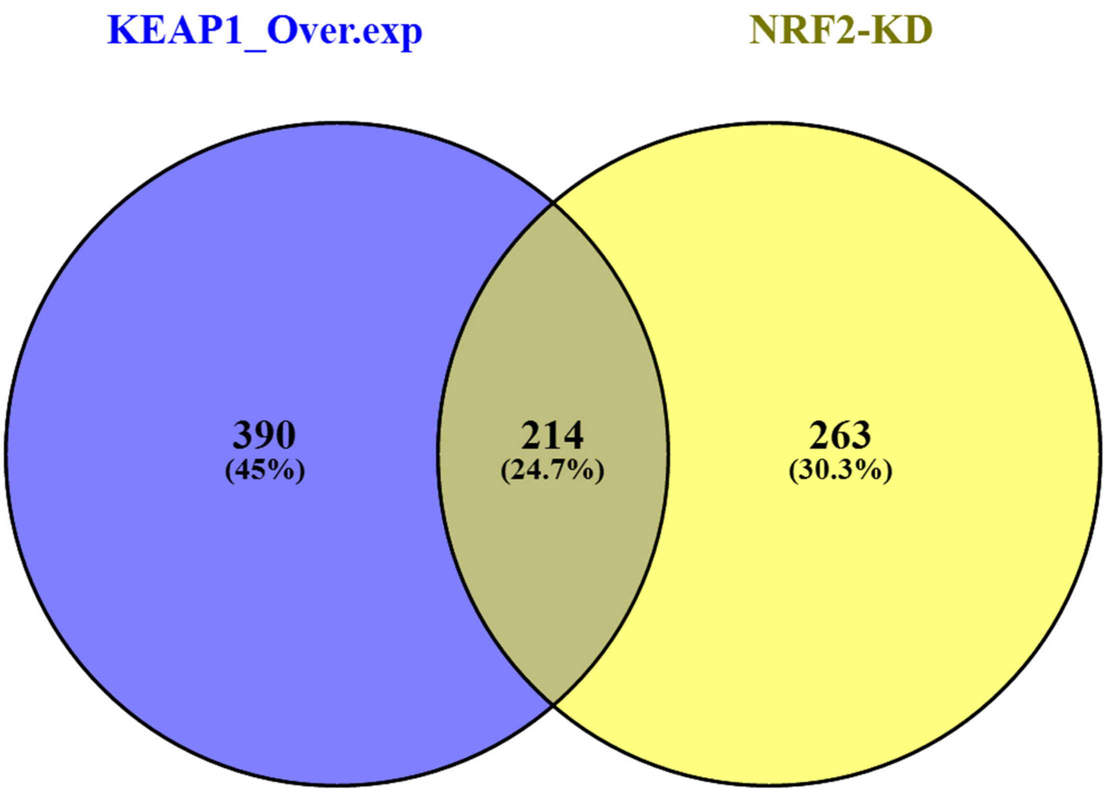

Figure 2: Venn diagram showing the genes overlapping between common NRF2-regulated genes identified from the combinatorial analysis of KEAP1 overexpression (KEAP1_Over.exp) and NRF2-knockdown (NRF2-KD) downregulated gene microarray data with fold change >1.5.

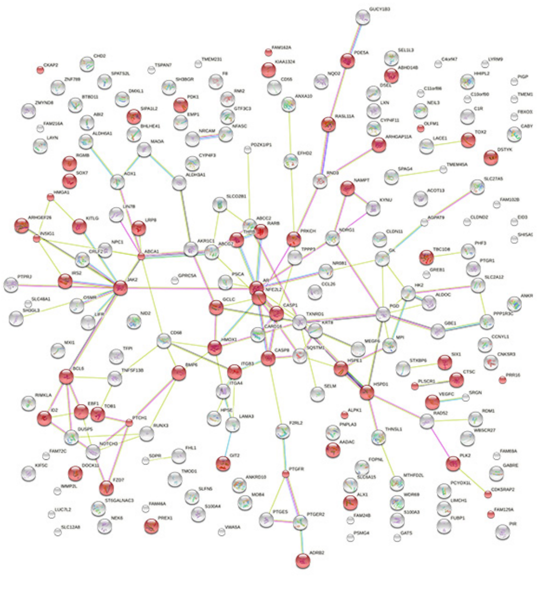

Figure 3: Protein-protein interaction network of downregulated genes identified from combinatorial analysis of both KEAP1-overexpression and NRF2-KD downregulated genes in A549 cells using STRING v10. Red, genes involved in GO enrichment: GO.0009893-positive regulation of metabolic process.

Supplementary Table 1: List of down regulated genes in KEAP1 overexpression A549 cells (FC>1.5).

See Supplementary File 1

Supplementary Table 2: Functional annotation of KEAP1 overexpression microarray down regulated genes in A549 cells.

See Supplementary File 2

Supplementary Table 3: List of down regulated genes in NRF2 KD A549 cells (FC>1.5).

See Supplementary File 3

Supplementary Table 4: Functional annotation of NRF2 KD microarray down regulated genes in A549 cells.

See Supplementary File 4

Supplementary Table 5: List of down regulated genes in both KEAP1 overexpression and NRF2 KD A549 cells (FC>1.5).

See Supplementary File 5

Supplementary Table 6: List of genes identified in GO_BP term- "small molecule metabolic process".

See Supplementary File 6

Supplementary Table 7: List of NRF2-ARE/ARE like sequences in genes involved in metabolism.

See Supplementary File 7

Supplementary Table 8: List of human qRT PCR primers used for validation.

See Supplementary File 8
